# Supplementary material for: Effects of Andrographolide-Loaded Nanostructured Lipid Carriers on Growth, Feed Efficiency, and Resistance to Streptococcus agalactiae in Nile Tilapia (Oreochromis niloticus)
Source: Animals (Basel). 2025 Jul 17;15(14):2117. doi: 10.3390/ani15142117 (PMC12291780; doi:10.3390/ani15142117)
Supplement: Supplementary file 1 [file animals-15-02117-s001.zip › animals-3677156-supplementary.pdf]

Supplementary file

Table S1. Paired t-test results comparing growth performance and feed utilization parameters (Final Weight [FW], Weight Gain [WG], Specific Growth Rate [SGR], Feed Conversion Ratio [FCR], and Protein Efficiency Ratio [PER]) of Nile tilapia between day 30 and day 60 across different dietary treatments (Control, andrographolide (AND), andrographolide loaded nanostructured lipid carriers (AND-NLC) and nanostructured lipid carriers (NLC)).

Parameters	t	df	*p*
FW			
Control	-10.177	58	0.001
AND	-12.297		0.001
AND-NLC	-10.489		0.001
NLC	-9.378		0.001
WG			
Control	-10.177	58	0.001
AND	-12.297		0.001
AND-NLC	-10.489		0.001
NLC	-9.378		0.001
SGR			
Control	1.472	58	0.073
AND	1.526		0.066
AND-NLC	-0.421		0.338
NLC	2.621		0.006
FCR			
Control	0.576	58	0.284
AND	1.778		0.040
AND-NLC	3.265		0.001
NLC	-0.156		0.438
PER			
Control	-10.177	58	0.001
AND	-12.297		0.001
AND-NLC	-10.489		0.001
NLC	-9.378		0.001
PER			
Control	-0.845	15	0.206
AND	-6.043		0.001
AND-NLC	-2.175		0.023
NLC	-2.770		0.007
